# Is plant evolutionary history impacting recruitment of diazotrophs and *nifH* expression in the rhizosphere?

**DOI:** 10.1038/srep21690

**Published:** 2016-02-23

**Authors:** Marie-Lara Bouffaud, Sébastien Renoud, Yvan Moënne-Loccoz, Daniel Muller

**Affiliations:** 1 Université de Lyon, F-69622, Lyon, France; 2 Université Lyon 1, Villeurbanne, France; 3 CNRS, UMR5557, Ecologie Microbienne, Villeurbanne, France

## Abstract

Plant evolutionary history influences the taxonomic composition of the root-associated bacterial community, but whether it can also modulate its functioning is unknown. Here, we tested the hypothesis that crop diversification is a significant factor determining the ecology of the functional group of nitrogen-fixing bacteria the rhizosphere of Poaceae. A greenhouse experiment was carried out using a range of Poaceae, i.e. four *Zea mays* varieties (from two genetic groups) and teosinte (representing maize’s ancestor), sorghum (from the same *Panicoideae* subfamily), and wheat (from neighboring *Pooideae* subfamily), as well as the dicot tomato as external reference. Diazotroph rhizosphere community was characterized at 21 days in terms of size (quantitative PCR of *nifH* genes), composition (T-RFLP and partial sequencing of *nifH* alleles) and functioning (quantitative RT-PCR, T-RFLP and partial sequencing of *nifH* transcripts). Plant species and varieties had a significant effect on diazotroph community size and the number of *nifH* transcripts per root system. Contrarily to expectations, however, there was no relation between Poaceae evolutionary history and the size, diversity or expression of the rhizosphere diazotroph community. These results suggest a constant selection of this functional group through evolution for optimization of nitrogen fixation in the rhizosphere.

Nitrogen is essential for plant growth and health. However, it is not always bioavailable in soil, as most nitrogen is present as N_2_ in soil porosity or as part of humic compounds. Amongst prokaryotes, diazotrophs can facilitate plant nitrogen nutrition through biological nitrogen fixation, which corresponds to the reduction of atmospheric nitrogen (N_2_) to ammonia (NH_3_). This occurs in agricultural as well as in natural environments, and amounts to 120 million tons of N are fixed annually on earth^1^. Biological nitrogen fixation is only performed by certain bacterial and archaeal taxa, and all rely on the *nifH* gene encoding the iron protein subunit of the nitrogenase, a gene widely used as marker to examine nitrogen fixation in various environments^2^,^3^,^4^. This gene is highly conserved among diazotrophs, and its phylogeny is largely correlated to 16S rRNA phylogeny^5^, making it a marker suitable to assess complex diazotrophic communities.

In terrestrial systems, diazotrophic community size and/or composition can vary according to soil type^3^,^6^,^7^,^8^, soil management^9^, season^7^, fertilization rate^2^,^10^, plant presence/absence^2^,^11^,^12^, plant growth stage^3^,^12^, plant species^13^,^14^,^15^,^16^ or plant varieties^2^,^17^. Some of these factors may also influence the composition of the bacterial community actually expressing *nifH* and/or the amount of *nifH* transcripts, even if the latter does not necessarily vary to a large extent^18^,^19^,^20^,^21^.

Differences in root-associated nitrogen-fixing bacterial communities between plant genotypes may involve differences in (i) root system development^22^ and thus bacterial root-colonization sites, (ii) nutrient uptake by roots^23^ and therefore rhizosphere depletion of minerals including nitrate and ammonium, and (iii) root exudation patterns^24^ and thus the flux of carbon sources and energy available to support BNF. However, plant genetic properties accounting for these plant phenotypic differences are not well understood, and those determining differences in root-associated functional microbial groups are even less understood. Evolutionary processes, whether corresponding to natural selection and/or human selection in agrosystems, have resulted in a wide range of plant genotypes on earth^25^ and have had a strong influence on root traits^23^,^26^,^27^,^28^. Recently, it was shown that the evolutionary history of Poaceae grown in a same soil was a significant factor determining the taxonomic composition of the total rhizobacterial community^29^. This was also the case at the level of several bacterial genera containing nitrogen-fixing species or strains, raising the possibility that root-associated microbial functional groups, such as nitrogen-fixing bacteria, may also be influenced by the evolutionary history of Poaceae. On this basis, our hypothesis was that plant evolutionary history can be a significant factor influencing the interaction of roots with microbial functional groups.

The objective of this study was to assess whether a relation exists between Poaceae evolution and root-associated diazotroph community of the resulting plant genotypes. To this end, we compared Poaceae both at infraspecific (four lines from two contrasted genetic groups of maize and one teosinte representing maize’s pre-domestication ancestor) and interspecific levels (one sorghum from maize’s *Panicoideae* subfamily, and one wheat from neighboring *Pooideae* subfamily) in a same soil under greenhouse conditions. A dicot (tomato) was also included as non-*Poaceae* external reference. The diazotroph community was characterized at 21 days in terms of size (*nifH* quantitative PCR), composition (*nifH* T-RFLP and partial sequencing of *nifH* gene) and functioning (quantitative RT-PCR, T-RFLP and partial sequencing of *nifH* transcripts). For selected treatments, the analyses were also carried out with better-established plants (i.e. at 42 days after sowing) as well as with 21-day-old plants grown in soil from a neighboring field (from the same soil type but under permanent meadow instead of maize monocropping), because in both cases rhizobacterial community structure and taxonomic composition differed^29^.

## Results

### Size of diazotroph communities

**Figure 1:**
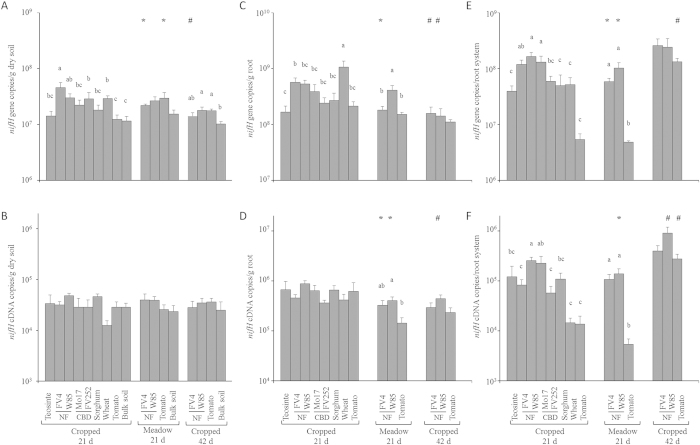
Quantification of *nifH* genes and transcripts in bulk soil and rhizosphere of Poaceae genotypes by real-time PCR and RT-PCR, respectively. Gene copy numbers are shown in (**A,C,E**) and transcript numbers in (**B,D,F**). Statistical analyses were performed independently at 21 days in cropped soil, at 21 days in meadow soil and at 42 days in cropped soil, using ANOVA and Fisher LSD tests (*P* < 0.05; results shown with letters a to (**D**). For FV4, W85, tomato and bulk soil, two-way ANOVA and Fisher LSD tests (*P* < 0.05) were also performed to compare treatments according to past soil management or sampling time, and differences with the same genotype at 21 days in cropped soil are indicated by symbols *and #respectively.

The total nitrogen-fixing bacterial community amounted to 1.0–4.6 × 10^7^ *nifH* gene copies per g of soil (Fig. 1A), and for plant treatments it meant 1.1–11.0 × 10^8^ *nifH* copies per g of root (Fig. 1C) or 4.8–260 × 10^8^ *nifH* copies per root system (Fig. 1E). At 21 days in cropped soil, diazotroph community size per g of soil was higher in the presence of plant for all maize lines and for wheat (Fig. 1A). This was also the case for 2 of 3 plant genotypes at 42 days in the same soil, whereas differences were not significant at 21 days in meadow soil.

When expressed per g of root, the diazotroph community size at 21 days in cropped soil did not differ for maize line FV252, teosinte, sorghum and tomato, whereas wheat and the two Northern Flint maize lines gave higher levels (Fig. 1C). At 21 days, differences (but not the same as in cropped soil) between plant genotypes were also found in meadow soil, whereas these differences were not significant at 42 days in cropped soil.

Since root system development varied according to plant genotype (as well as sampling time and past soil management for maize line FV4) (Figure S1A), diazotroph community size was also considered per root system. When doing so (Fig. 1E), diazotroph community at 21 days in cropped soil did not differ in size for teosinte, wheat, sorghum, whereas its size was higher for maize lines W85 and Mo17 and lower for tomato. *nifH* copy number was also lower for tomato than for maize at 21 days in meadow soil, whereas differences were not significant at 42 days in cropped soil.

Overall, differences in diazotroph community size were found according to plant genotype, sampling time or past soil management. Even though differences occurred between plant genotypes, more closely related plant genotypes did not necessarily display a diazotroph community closer in size.

### nifH transcript levels in diazotroph communities

The number of *nifH* transcripts from the nitrogen-fixing bacterial community reached 1.3–4.8 × 10^4^ *nifH* cDNA copies per g of soil (Fig. 1B), and for plant treatments it corresponded to 1.4–8.9 × 10^5^ *nifH* cDNA copies per g of root (Fig. 1D) or 5.4–910 × 10^3^ *nifH* cDNA copies per root system (Fig. 1F). At 21 days in cropped soil, the number of *nifH* transcripts per g of soil was similar in all treatments (Fig. 1B). This was also the case in the same soil when considering the other sampling (42 days), or at 21 days in meadow soil.

When focusing on plant treatments and expressing data per g of root, regardless of the amount of rhizosphere soil (Figure S1B), there was again no difference between genotypes in the number of *nifH* transcripts in cropped soil at 21 or at 42 days (Fig. 1D). In meadow soil at 21 days, however, the number of *nifH* transcripts per g root was higher for the two maize lines than for tomato.

When integrating root system size, the number of *nifH* transcripts (i.e. expressed per root system) at 21 days in cropped soil was higher for maize lines W85 and Mo17 than for maize lines FV4 and FV252, wheat and tomato (Fig. 1F). At 21 days in meadow soil, the number of *nifH* transcripts per root system was again higher for maize line W85 than for tomato, but this time also maize line FV4 gave higher values than tomato. There was no difference in cropped soil at 42 days, when tomato root system was larger.

Overall, differences in *nifH* transcript number were mainly found when comparing plant treatments using data expressed per root system (i.e. at the scale of entire rhizospheres), but plant genotypes from the same maize group or species did not give values more similar than did genotypes from different species.

### Diversity of diazotroph communities

**Figure 2:**
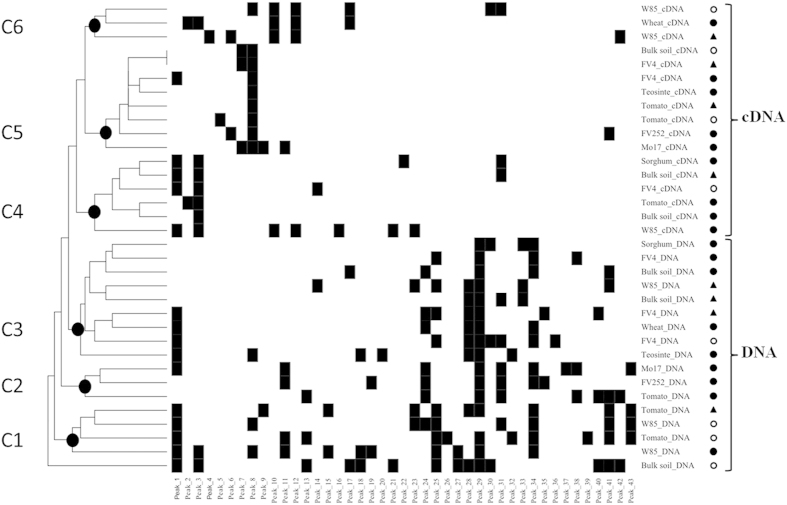
Comparison of T-RFLP profiles from bulk soil and rhizosphere of Poaceae genotypes based on *nifH* DNA and cDNA samples in cropped soil at 21 days (black circles) and 42 days (white circles) after sowing, and in meadow soil at 21 days (black triangles). The analysis was based on presence/absence of T-RFs in each treatment, using Euclidean distance matrix and complete linkage clustering. The two T-RFs evidenced by in-silico T-RFLP of *nifH* qPCR and qRT-PCR products from W85 maize correspond to peaks 1 and 2.

T-RFLP of bulk soil and rhizosphere treatments revealed a total of 43 distinct T-RFs (from 30 bp to full 350-bp polF/polR amplicon size), of which 21T-RFs were found only in DNA samples, 9 in cDNA samples and 13 in both (Fig. 2). With DNA samples, 3T-RFs were evidenced in at least 10 of 17 treatments, 8 in 6–9 treatments, 18 in 2–5 treatments, and 8 in only 1 treatment. With cDNA samples, 2T-RFs were evidenced in 6–9 treatments, 8 in 2–5 treatments, and 12 in only 1 treatment. The genetic distance between samples was higher (meaning a lower profile variability) for DNA samples (0.53 ± 0.01) than for cDNA samples (0.46 ± 0.01).

Clustering of all treatment combinations based on T-RFLP data gave 3 clusters (C1–C3) as well as, separately, the bulk soil sample from cropped soil at 42 days for DNA samples, and 3 other clusters (C4–C6) for cDNA samples (Fig. 2). When considering past soil management, cropped soil samples clustered with meadow soil samples for bulk soil DNA (in C3), maize FV4 DNA (in C3) and maize FV4 cDNA (in C5), which was not the case for bulk soil cDNA (C4 vs C5), maize W85 DNA (C1 vs C3) and cDNA (C4 vs C6), or tomato DNA (C2 vs C3) and cDNA (C4 vs C5). Cropped soil samples at 21 days clustered with the ones at 42 days for maize FV4 DNA (in C3) and maize W85 DNA (in C1), but not for the other DNA samples or any of the cDNA samples, even in the case of bulk soil. Against this background, the two maize lines FV252 and Mo17 of the genetic group Corn Belt Dent clustered together both for DNA (in C2) and cDNA (in C5). However, it was not the case with the two Northern Flint maize lines (for DNA or cDNA), and overall the four maize lines were split in all three DNA clusters and all three cDNA clusters. No particular relation was found either when adding successively the fifth *Zea mays* genotype (teosinte), sorghum, and wheat, regardless of whether cDNA or DNA was considered.

Sequencing the qPCR and qRT-PCR products from the W85 sample studied gave 70 and 180 partial *nifH* sequences, respectively, which gave 14 clades and 3 single sequences (Figure S2). Six of the 14 clades contained *nifH* sequences from known taxa, i.e. *Burkholderia*/*Curvibacter* (clade 2), *Ideonella*/*Azohydromonas*/*Ensifer* (clade 8), *Rubrivivax* (clade 9), *Azorhizobium/Bradyrhizobium* (clade 10), *Azospirillum* (clade 12) and *Rhizobium*/*Sinorhizobium* (clade 13), whereas the eight others did not. Except for clade 10, each clade included both DNA and RNA sequences. In silico T-RFLP analyses of the 250 *nifH* DNA and cDNA sequences gave only 2 distinct T-RFs, which correspond to T-RFs actually obtained by T-RFLP of *nifH* qPCR and qRT-PCR products.

### Relationship between Poaceae phylogeny and diazotroph community

**Figure 3:**
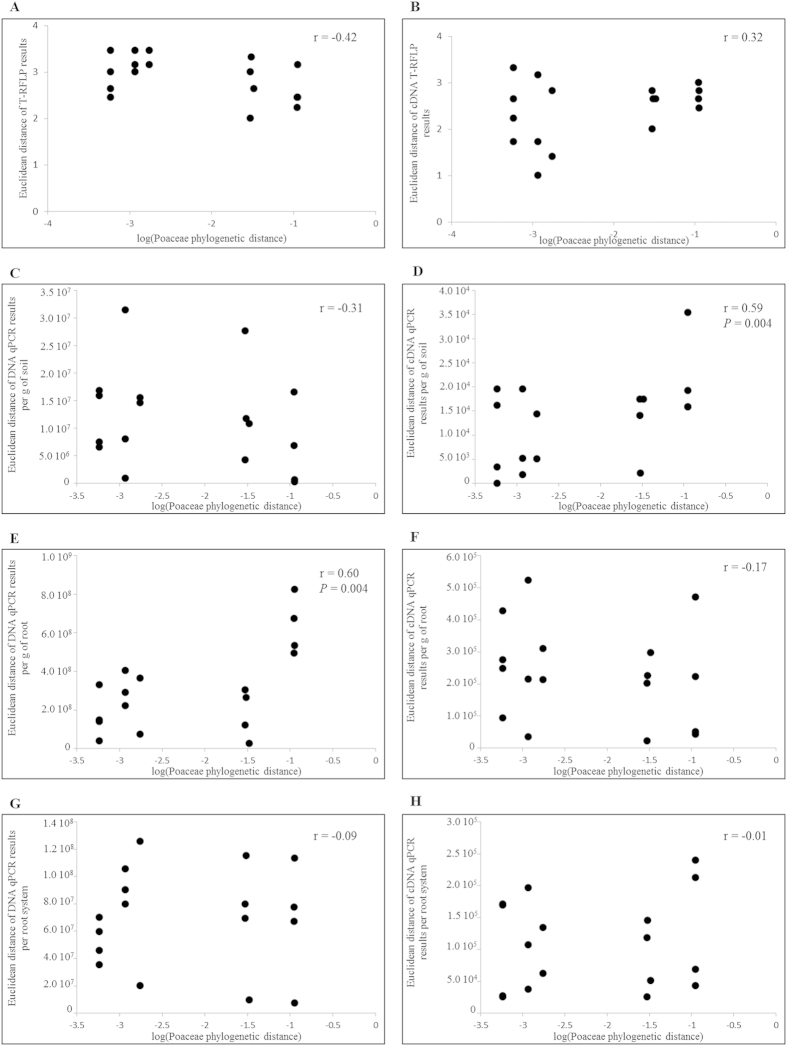
Pairwise comparisons (n = 18) of plant phylogenetic distance between Poaceae (X axis) with the corresponding distance between their root-associated diazotroph communities (Y axis). *nifH* DNA data are shown to the left and *nifH* cDNA data to the right, and they correspond to results of T-RFLP (**A,B**), quantitative PCR or RT-PCR expressed per g of soil (**C,D**), per g of root (**E,F**) or per root system (**G,H**). Distances were calculated two by two, using Kimura 2 parameter model for plant phylogeny and Euclidean distance for diazotrophic community. The Pearson correlation coefficient and its probability level (when *P* < 0.05) are indicated.

For a more formal appraisal of the relationship between diazotroph community and Poaceae phylogeny, correlation analysis was carried out between pairwise Poaceae phylogenetic distances and Euclidean distances derived from their root-associated diazotroph communities at 21 days in cropped soil (Fig. 3). A significant correlation was found when considering *nifH* gene copy number per g of root (r = 0.60, *P* = 0.004, n = 18; Fig. 3E) but not per g of rhizosphere soil or per root system (Fig. 3C,G), as well as *nifH* transcript number per g of rhizosphere soil (r = 0.59, *P* = 0.004, n = 18; Fig. 3D) but not per g of root or per root system (Fig. 3F,H). No significant correlation was obtained when considering diazotroph community diversity, regardless of whether data were derived from DNA or cDNA T-RFLP analysis (Fig. 3A,B).

### Relationship between plant development and diazotroph community

A significant correlation was found when assessing plant phylogenetic distance against differences in plant development (in terms of shoot or root biomass) but not rhizosphere size (Table 1). Differences in the number of *nifH* gene copies correlated with differences in (i) shoot and root biomass (when expressed in copies per g of root), (ii) root biomass and rhizosphere mass (when expressed in copies per g of rhizosphere soil) and (iii) rhizosphere mass (when expressed in copies per root system). In addition, differences in the number of *nifH* transcripts correlated with differences in shoot biomass, root biomass and rhizosphere mass when expressed in copies per root system. Conversely, there was no correlation between (i) differences in shoot biomass, root biomass and rhizosphere mass and (ii) distances between T-RFLP profiles for *nifH* genes or transcripts.

## Discussion

In this study, the impact of plant evolutionary history on root-associated diazotroph community size, *nifH* transcript number and genetic diversity was studied using molecular approaches based on established *nifH* primers^9^,^20^. The choice of primers is critical to capture the diversity of a functional group. Different *nifH* primer sets were designed in the last two decades and their potential to recover the majority of known *nifH* alleles was evaluated several times, with contradictory results^30^. When assessed *in silico*, primers such as the Zf/Zr pair^31^ presented a higher theoretical recovery of *nifH* diversity than the polF/polR primers used here^32^, but polF/polR display higher performance^9^,^20^ *in vitro* than *in silico* and can be used directly for qPCR (unlike others that are less specific^9^,^20^,^30^ and could require nested PCR^31^).

In the case of nitrogen-fixing rhizobia, the interaction with the plant (Fabales) leads to nodule structural differentiation^34^ and provides mutual benefits to the partners^35^. Most other plant families do not engage in this type of symbiosis, but in soil a large range of free-living microorganisms interact with plant roots, providing here also mutual benefits^36^,^37^,^38^. Although no partner differentiation takes place, it makes sense that during evolution, modifications of root phenotypic properties facilitating bacterial nitrogen fixation and thereby the supply of available nitrogen to the plant could have been selected.

On the one hand, many plant-beneficial free-living bacteria can benefit plants, but with contrasted efficacy according to plant species or varieties^39^,^40^. On the other hand, Poaceae evolutionary history is a factor influencing root selection of soil bacteria and thus total rhizobacterial community composition^29^. This concerns in particular many genera and species of nitrogen-fixing bacteria from the orders *Rhodospirillales*, *Burkholderiales* and *Enterobacteriales*. However, even though a significant relation was found in Bouffaud *et al*.^29^ between total rhizobacterial community composition and the phylogeny of the same Poaceae genotypes used in this study, no relation was found here when considering diazotroph community composition (Fig. 3). One such correlation was found with *nifH* gene copy number and another with *nifH* transcript number, but their relevance is probably limited because (i) the former was significant when *nifH* gene copy number was expressed per g of root but not per g of rhizosphere soil and the latter when *nifH* transcript number was expressed per g of rhizosphere soil but not per g of root, (ii) none of the two was significant when these community parameters were expressed per root system, which represents best the nitrogen fixation potential for plant individuals, and (iii) the two correlation coefficients were of modest level (≤0.60). On this basis, it can be concluded that Poaceae evolution did not influence significantly *nifH* community size, the quantity of *nifH* transcripts or genetic diversity of root-associated nitrogen-fixing community.

Two features might account for the lack of relation found between Poaceae evolution and root-associated nitrogen-fixing community. First, results showed *nifH* bacteria were well present and expressed *nifH* in bulk soil, which means that presence of the plant was not required for their establishment and functioning (Fig. 1), thereby limiting the potential impact of plant features on the diazotroph community. Second, the relation between Poaceae evolution and rhizobacterial community composition found by Bouffaud *et al*.^29^ with the same seven plant genotypes was relevant for only one third (91 probes of 298) of bacterial taxa, which could mean that the housekeeping plant markers that were used did not reflect sufficiently the whole scope of plant traits relevant for plant-bacteria interactions. Another third (51 probes) of the 150 most discriminant 16S rRNA probes when comparing the taxonomic composition of the total rhizobacterial community^29^ targeted diazotrophic bacteria. The signal of 31 of these 51 diazotroph probes correlated in fact with maize/Poaceae genetic distances (e.g. for *Azospirillum*, *Gluconobacter*, *Paenibacillus*, etc.). However, it was not the case for the 20 other probes (e.g. for *Bradyrhizobium*, *Devosia*, etc.), which did not enable to yield a significant relation at the scale of the whole diazotrophic functional group.

Although Poaceae evolution was not a significant factor, an impact of plant genotype was yet found on diazotroph community size, expression or diversity. The impact on qPCR and qRT-PCR levels was rather moderate in magnitude, in accordance with previous studies showing differences usually not exceeding one log for DNA or cDNA copy number between samples from different areas or management practices^15^,^20^,^21^,^41^,^42^,^43^. This could suggest that a basal level of diazotrophic community size and expression always occurs (as discussed above for bulk soil) and is not extensively affected by environmental factors. In comparison, shifts in diazotrophic community populations occur readily^20^, including when comparing plant genotypes as found here (Fig. 2) and in previous work^2^,^12^,^14^,^17^. However, there was no apparent pattern of diazotrophic community structuration when considering genotypic groups defined within the maize species or across different plant species. Against this background, significant relations were found between plant development parameters and *nifH* gene or transcript numbers, suggesting that plant development could be a more important factor than plant evolutionary history, and that occasional relations evidenced between Poaceae evolutionary history and *nifH* parameters probably involved Poaceae evolutionary history effects on plant development parameters.

In conclusion, this study showed that the effect of Poaceae evolutionary history on the root-associated diazotroph community was not significant, contrarily to expectations derived from the observation^29^ that plant evolutionary history did influence the taxonomic composition of the entire rhizobacterial community of Poaceae.

## Materials and methods

### Plant genotypes and greenhouse experiment

Plant genotypes included four cultivated maize (*Zea mays* L.), i.e. the inbred lines FV4, W85 (group Northern Flint), FV252 and Mo17 (group Corn Belt Dent) (provided by INRA, St Martin d’Hynx, France), one teosinte (*Zea mays* ssp. *parviglumis*; provided by UNAM, Cuernavaca, Mexico), one sorghum (*Sorghum bicolor* L. cv. Arprim; Semences de Provence, Fourques, France), one wheat (*Triticum aestivum* L. cv. Fiorina; provided by AgroScope, Changins, Switzerland), and one tomato (*Solanum lycopersicum* L. cv. Marmande; Vilmorin, La Ménitré, France) used as non-Poaceae, external reference.

We used the plant experiment carried out by Bouffaud *et al*.^29^. Plants were grown in loamy topsoil (sieved at 6 mm) collected in September 2009 from two adjacent fields (luvisols) located at La Côte Saint-André near Lyon (France). One is a maize-monocropping field (topsoil: clay 15.9%, silt 41.4%, sand 42.7%, organic matter 2.3%, pH (water) 7.3, N 1.6 g kg^−1^) and the other a permanent meadow (topsoil: clay 14.9%, silt 44.6%, sand 40.5%, organic matter 5.5%, pH (water) 6.0, N 3.2 g kg^−1^). Briefly, seeds were surface-disinfected and sown in 3-dm^3^ pots containing 2.5 kg soil (to obtain one seedling per pot), and the pots (including non-planted pots) were placed (randomized blocks; 5 pots per treatment) in a greenhouse. Sampling was carried out at 21 days in cropped soil for each treatment. In addition, maize lines FV4 and W85 (group Northern Flint) and tomato were also sampled at 21 days in meadow soil and at 42 days in cropped soil. Each root system was dug up, shaken vigorously (to eliminate soil loosely adhering to the roots), frozen (along with closely-adhering soil) in liquid nitrogen and lyophilized. Root and rhizosphere soil were separated, and each stored at −20 °C (giving 0.1–0.7 g root samples and 0.5–6 g rhizosphere soil samples per plant). In addition, bulk soil was sampled in non-planted pots, at 21 (both soils) and 42 days, frozen, lyophilized and stored at −20 °C (giving 5 g samples).

### Extraction of soil DNA and RNA

We used total nucleic acids that were extracted by Bouffaud *et al*.^29^, from 0.5 g of lyophilized rhizosphere or bulk soil using a protocol derived from Burgmann *et al*.^44^. Briefly, 0.5 g rhizosphere or bulk soil, 0.5 g zirconium beads (VWR, Fontenay-sous-Bois, France), 0.5 ml extraction buffer (5% hexadecyltrimethylammonium bromide, 1 mM 1,4-dithio-DL-threitol, in a 0.12 M phosphate buffer [pH 8]) were processed in a bead beater (TissueLyser II Retsch; Qiagen, Courtaboeuf, France) for 90 s at 30 m s^−1^. After 10 min centrifugation at 16,000 *g*, supernatants were extracted two times with phenol-chloroform-isoamyl alcohol (24:24:1 v/v/v), and then once with chloroform-isoamyl alcohol (24:1 v/v). Nucleic acids were precipitated overnight with potassium acetate (3 M, pH 4.8) and absolute ethanol at −20 °C. After centrifugation 30 min at 16,000 *g*, pellets were washed with 70% ethanol, and dissolved in 100 μl RNase- and DNase-free water (giving 50–100 ng nucleic acids per μl).

### cDNA synthesis by reverse transcription

Here, to obtain DNA-free RNA, 20 μl of nucleic acid solution were digested at room temperature with 4 U of DNase I (Invitrogen, Cergy Pontoise, France) in 1 × DNase I reaction buffer, and RNA was purified using RNeasy Mini kit (Qiagen) following manufacturer’s instructions. Another step of DNA digestion was performed using the protocol described above to remove remaining traces of DNA, and the reaction was stopped by incubating 10 min at 65 °C in presence of 1 μl of 25 mM EDTA.

Reverse transcription (RT) was performed on 8 μl of the resulting purified RNA extract using Omniscript reverse transcription kit (Qiagen; following the manufacturer’s instructions). The reaction was carried out 90 min at 37 °C. Inactivated was done at 95 °C (10 min) and cDNA was stored at −20 °C.

### Quantitative PCR and quantitative RT-PCR

When selecting *nifH* primers, PolF/PolR^9^ were preferred over Zf/Zr^31^ because the latter proved ineffective for quantitative PCR (qPCR), giving non-specific products and smeared bands on gels^4^,^9^, and PolF/PolR is among recommended primers to capture *nifH* diversity efficiently^19^,^20^. Here, the amounts of *nifH* genes and transcripts (from rhizosphere or bulk soil) were estimated by qPCR and quantitative RT-PCR (qRT-PCR), after development of a real-time protocol based on the primers polF/polR^9^. The reaction was carried out in 20 μl containing 4 μl of PCR grade water, 2 μl of each primer (final concentration 0.50 μM), 10 μl of LightCycler-DNA Master SYBR Green I master mix (Roche Applied Science, Meylan, France) and 2 μl of sample DNA or cDNA (in triplicate for each of five repetitions per treatment). The cycling program included 10 min incubation at 95 °C, followed by 50 cycles of 95 °C for 15 s, 64 °C for 15 s and 72 °C for 10 s. Amplification specificity was studied by melting curve analysis of the PCR (and RT-PCR) products, performed by ramping the temperature to 95 °C for 10 s and back to 65 °C for 15 s, followed by increases of 0.1 °C s^−1^ up to 95 °C. Melting curve calculation and determination of Tm values were performed using the polynomial algorithm function of LightCycler Software v.1 (Roche Applied Science).

Genomic DNA from *Azospirillum lipoferum* 4B (whose genome contains one *nifH* copy) was used to generate standard curves, after dilution from 5 × 10^−9^ to 5 × 10^−15^ g DNA μl^−1^ (in triplicate). Sterile water was used as negative control for DNA amplification, and DNAse treated RNA before reverse transcription was used as negative control for cDNA amplification. PCR efficiency was calculated from standard curves according to the equation E = 10^(−1/slope)^. The five samples for each rhizosphere or bulk soil treatment were analyzed. Results in g μl^−1^ were converted in number of *nifH* copies using the following formula (assuming an average of 660 g mol^−1^ per base pair): number of copies = [DNA (g)× Avogadro’s number (molecules mol^−1^)]/[number of DNA base pairs in *nifH* fragment ×660 (g mol^−1^)]. The resulting numbers were expressed (i) per g of root, (ii) per root system, and (iii) per g of soil.

### T-RFLP analysis

For one replicate (individual plant or bulk soil sample) per treatment, *nifH* DNA and cDNA were amplified using forward primer polF^9^ 5′-labeled with 6-FAM and reverse primer polR. PCR reaction was carried out in 50 μl containing 1 × buffer, 1 μM of each primer (Invitrogen), 2.5 mM of MgCl_2_, 1.75 U of Expand High Fidelity Taq polymerase (Roche Applied Science) and 2 μl of DNA or cDNA template. An initial denaturation at 94 °C for 2 min was followed by 30 cycles of 45 s denaturation at 94 °C, 30 s annealing at 55 °C and 30 s extension at 72 °C, followed by a final extension for 5 min. PCR products were purified using PCR purification kit (Macherey-Nagel, Hoerd, France). For T-RFLP, 500 ng of PCR product were digested using HaeIII (Fermentas, Villebon sur Yvette, France) 3 h at 37 °C and separated on automated sequencer ABI 3730XL (Applied Biosystems, Villebon sur Yvette, France). The number of individual terminal restriction fragments (T-RFs) was determined using GeneMapper v4.1 software (Applied Biosystems), with a detection limit of 50 relative fluorescence units.

### Partial nifH sequencing

To corroborate T-RFLP data, qPCR products from DNA and cDNA samples of maize line W85 were cloned and sequenced, using the polF/polR primer set in 50 μl containing ~50 ng of purified qPCR products. PCR was carried out as described above. The PCR products were purified and cloned in the pGEMs-T Easy Vector System kit (Promega, Charbonnières, France) and positive clones were sequenced (Biofidal, Vaulx-en-Velin, France). Nucleotide sequences were analyzed using the SeaView multiplatform graphical user interface^45^ (available at http://pbil.univ-lyon1.fr/) using MUSCLE^46^ (default parameters), and phylogenetic trees inferred using PhyML^46^ (version 3.0) with a GTR model of nucleotide substitution^47^. Reference *nifH* genes were retrieved using BLASTP^48^ at the National Center for Biotechnology Information (http://www.ncbi.nlm.nih.gov).

### Statistics

All analyses were done at *P* < 0.05, using R 2.10.1 software (http://www.r-project.org). First, treatments were compared concerning numbers of *nifH* genes and transcripts, using ANOVA and Fisher’s LSD tests, in each soil and at each sampling. Additionally, two-way ANOVA and Fisher’s LSD tests were performed to take into account past soil management at 21 days (i.e. treatment× past soil management) and sampling time effects in cropped soil (i.e. treatment × sampling + Error (samples/sampling).

Second, for T-RFLP data, treatments were compared based on peak presence/absence, using clustering analysis based on Euclidean distance and complete linkage clustering. One replicate (individual plant or bulk soil sample) was studied per treatment. For maize line W85, all five replicates were assessed, with similar results.

Third, Pearson correlation analysis was performed between (i) the phylogenetic distance between plant genotypes (Maximum Likelihood method, with the Kimura 2 parameter model, applied on three concatenated chloroplastic sequences i.e. gene *rps16* and the intergenic spacers *rps16-trnK* and *atpI-atpH*^29^) and (ii) the corresponding pairwise differences in *nifH* numbers (genes or transcripts, expressed without log transformation), T-RFLP profiles (genes or transcripts), plant biomass (shoots or roots) or weight of root-associated soil (i.e. rhizosphere soil), based on the Euclidean distance.

## Additional Information

**How to cite this article**: Bouffaud, M.-L. *et al*. Is plant evolutionary history impacting recruitment of diazotrophs and *nifH* expression in the rhizosphere? *Sci. Rep*. **6**, 21690; doi: 10.1038/srep21690 (2016).

## Supplementary Material

Supplementary Information

## Figures and Tables

**Table 1:** Pearson correlation (n = 18) and associated probability level in parenthesis (both in bold when *P* < 0.05) when comparing at 21 d in cropped soils (i) pairwise distance in dry weight of shoots, roots or rhizosphere soil and (ii) Poaceae phylogenetic distance or Euclidean distance for *nifH* genes or transcripts.

	Dry weight (g per plant)		
	Shoots	Roots	Rhizosphere soil
Poaceae phylogeny	**0.58** (**0.005**)	**0.55 (0.009)**	−0.07 (0.764)
*nifH* gene number			
Per g of rhizosphere soil	−0.47 (0.054)	**−0.55 (0.021)**	**−0.54 (0.024)**
Per g of root	**0.60 (0.012)**	**0.65 (0.005)**	0.09 (0.745)
Per root system	0.41 (0.101)	0.18 (0.489)	**0.66 (0.004)**
*nifH* transcript number			
Per g of rhizosphere soil	0.45 (0.068)	0.47 (0.058)	0.21 (0.415)
Per g of root	−0.13 (0.627)	−0.09 (0.733)	0.31 (0.232)
Per root system	**0.63 (0.007)**	**0.58 (0.014)**	**0.95 (<0.001)**
