# Supplementary material for: Is plant evolutionary history impacting recruitment of diazotrophs and nifH expression in the rhizosphere?
Source: Sci Rep. 2016 Feb 23;6:21690. doi: 10.1038/srep21690 (PMC4763242; doi:10.1038/srep21690)
Supplement: Supplementary Information [file srep21690-s1.pdf]

# SUPPLEMENTARY INFORMATION

Is plant evolutionary history impacting recruitment of diazotrophs and *nifH* expression in the rhizosphere?

Marie-Lara Bouffaud[1,2,3], Sébastien Renoud[1,2,3], Yvan Moënne-Loccoz[1,2,3], and Daniel Muller[1,2,3*]

[1] *Université de Lyon, F-69622, Lyon, France*

[2] *Université Lyon 1, Villeurbanne, France*

[3] *CNRS, UMR5557, Ecologie Microbienne, Villeurbanne, France*

[*]Correspondence: Daniel Muller, Fax: +33 472 43 12 23; E-mail: daniel.muller@univ-lyon1.fr

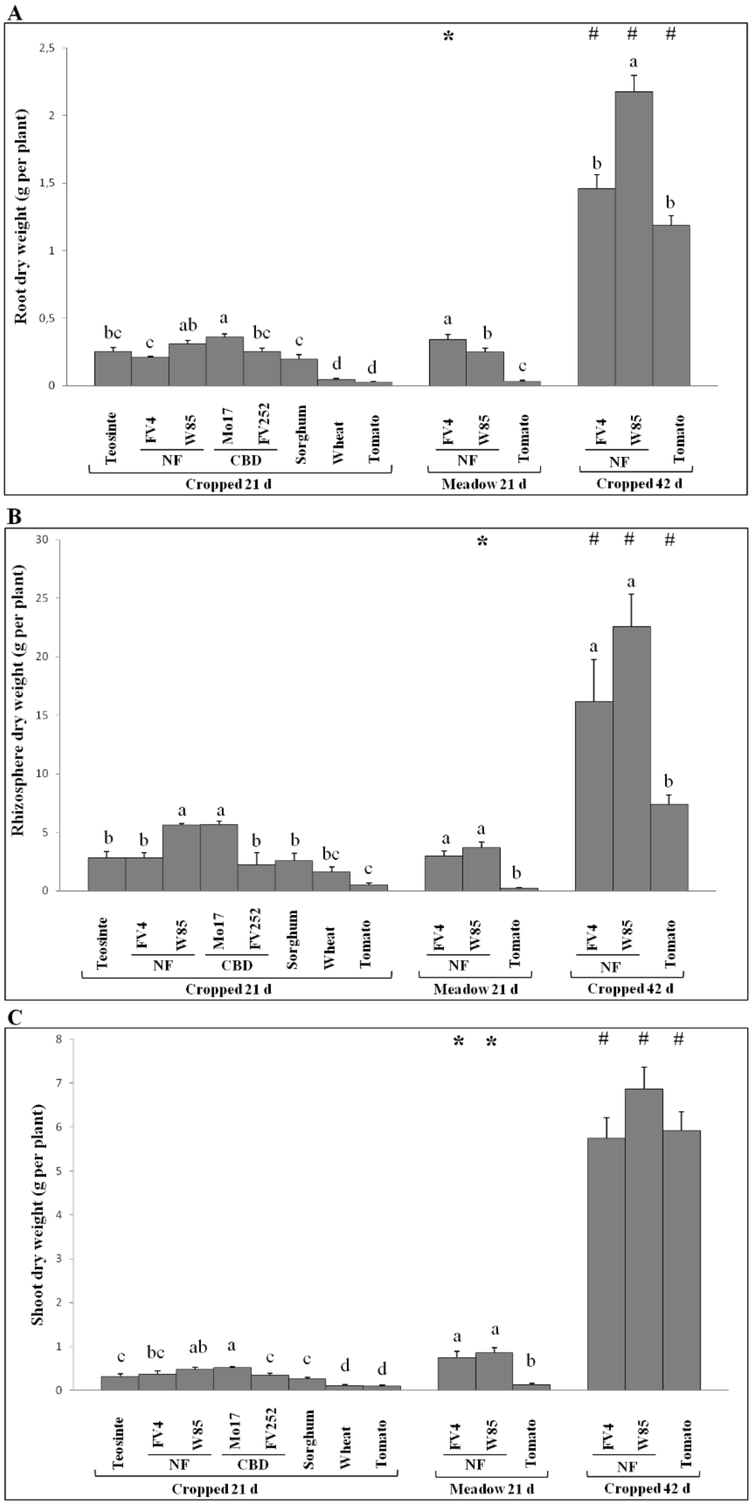


**Figure S1** Root (A), rhizosphere soil (B) and shoot (C) dry weight per plant. Statistical analyses were performed independently at 21 days in cropped soil, at 21 days in meadow soil and at 42 days in cropped soil, using ANOVA and Fisher LSD tests ($P < 0.05$; results shown with letters a to d). For maize lines FV4 and W85, tomato and bulk soil, two-way ANOVA and Fisher LSD tests ($P < 0.05$) were also performed to compare treatments according to past soil management or sampling time, and differences with the same genotype at 21 days in cropped soil are indicated by symbols * and #, respectively.

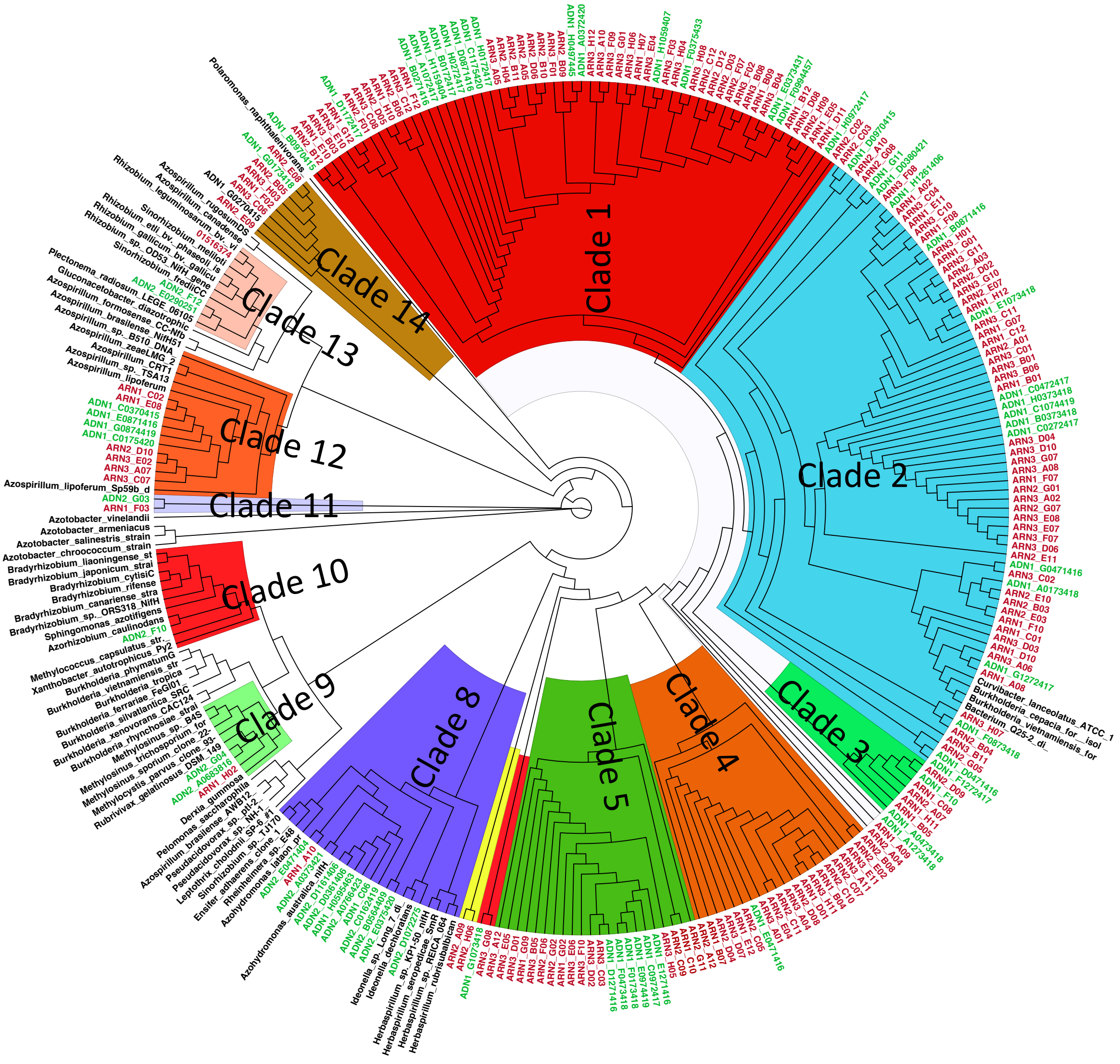


**Figure S2** Phylogenetic tree based on partial *nifH* DNA (red) and cDNA (green) sequences retrieved from the maize W85 sample studied at 21 days in cropped soil. The tree was constructed by maximum likelihood method. Representative *nifH* sequences are written in black, and the 14 clades identified are indicated.
